# Characterization and Evaluation of Injectable Biodegradable Polymer Multimodality Radiologic Markers in an In Vivo Murine Model

**DOI:** 10.1021/acs.biomac.1c01570

**Published:** 2022-03-31

**Authors:** Eliel Ben-David, Abraham J. Domb, Haixing Liao, Awanish Kumar, Issac Nissenbaum, Matthias Stechele, Peter Siman, Natalie Greenbaum, Naama Lev Cohain, S. Nahum Goldberg

**Affiliations:** † Faculty of Medicine, Hebrew University of Jerusalem, Jerusalem 9112102, Israel; ‡ The Department of Radiology, Shaare Zedek Medical Center, Jerusalem 9103102, Israel; § Institute of Drug Research, School of Pharmacy-Faculty of Medicine, The Hebrew University of Jerusalem, Jerusalem 9112102, Israel; ∥ Department of Ultrasonography, The First Affiliated Hospital of Guangzhou Medical University, Guangzhou 9112102, China; ⊥ Faculty of Medicine, Hebrew University of Jerusalem, Jerusalem 9112102, Israel; # The Department of Radiology, Hadassah-Hebrew University Medical Center, Jerusalem 91121, Israel; ∇ der Klinik und Poliklinik für Radiologie Ludwig-Maximilians-Universität München, Munich 81377, Germany; ○ Intragel, Nazareth Industrial Area, Wadi El Haj 13, P.O. Box 1252, Nazareth 17111, Israel

## Abstract

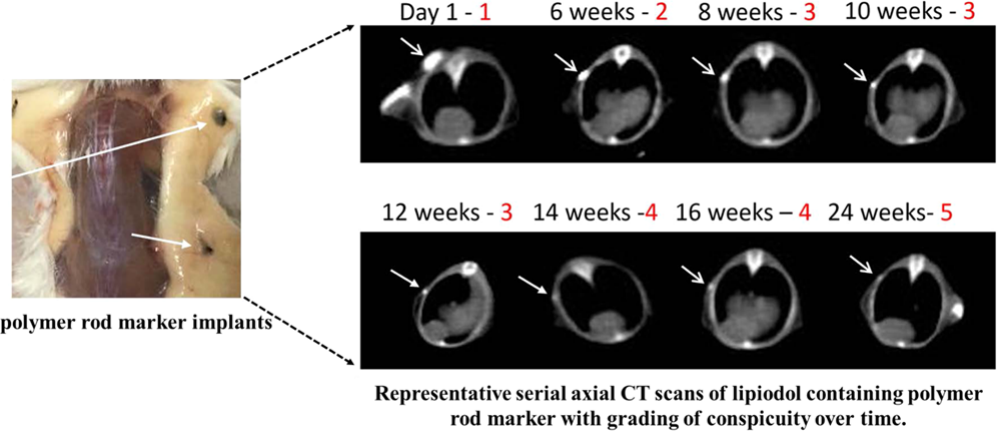

Biodegradable polymer clips as multidimensional soft tissue biopsy markers were developed with better biocompatibility and imaging features. Unlike the commercially available metallic biopsy markers, the developed polymer clips are temporary implants with similar efficacies as metal markers in imaging and detection and get absorbed within the body with time. Herein, we evaluate the degradation rate of three resorbable polymer-based marker compounds in an in vivo murine model. Three polymers, abbreviated as Polymer A (PLGA poly(lactic-*co*-glycolic acid)50:50), Polymer B (PLGA (poly(lactic-*co*-glycolic acid)) 75:25), and Polymer C (polycaprolactone (PCL)), mixed with 20% lipiodol and 0.2% iron oxide and a control polymer were implanted into nine mice, followed by CT and MRI imaging. Images were evaluated for conspicuity. Specimens were examined for tissue analysis of iodine and iron contents. Significant differences in polymer resorption and visualization on CT were noted, particularly at 8 weeks (*p* < 0.027). Polymers A, B, and C were visible by CT at 4, 6, and 8 weeks, respectively. All marker locations were detected on MRI (T1 and SWI) after 24 weeks, with tattooing of the surrounding soft tissue by iron deposits. CT and MR visible polymer markers can be constructed to possess variable resorption, with stability ranging between 4 and 14 weeks post placement, making this approach suitable for distinct clinical scenarios with varying time points.

## Introduction

Fiducial marker placement is often used to improve upon the accuracy of cutting-edge, state-of-the-art techniques, such as minimally invasive surgery,^1,2^ interventional procedures,^3^ and precision brachytherapy.^4^ Clinical scenarios include the need for marking of abnormal imaging findings or tumors, most commonly in the breast, to permit identification at surgery^5,6^ and the need for multiple three-dimensional markers for precise serial application of stereotactic radiation.^7^ Currently, these markers are most often metallic, making them permanent. Yet, permanency of the markers is often not required. For example, if management is surgical, markers are required for in situ identification for at most a range of a few weeks, and if radiation treatment is considered, the marker is usually required for only a 2–3-month period and most often not beyond a 6-month course of therapy. Nevertheless, retention of a foreign body beyond this time frame is extraneous and sometimes potentially undesirable. Particularly, it is crucial to minimize obscuring image artifacts, particularly in the region most likely to demonstrate recurrence, and metallic implants are known to exhibit a strong blooming artifact on MR (magnetic resonance) imaging.

To overcome this permanent nondegradable nature of metallic clips, slowly self-resorbing polymer-based markers have been developed.^8^ One early example is a product that incorporates a water-soluble polyethylene glycol-based hydrogel polymer, which is readily identified by ultrasound detection but poorly visualized on other modalities.^9^ Recently, a biodegradable implant has been designed and formulated incorporating clinically approved, commonly used contrast agents visible at both CT (computer tomography) and MR (magnetic resonance).^10^ In a preliminary in vitro and a short-term in vivo study, the visibility, shape, and degradation of these biodegradable implants containing Lipiodol (an X-ray contrast medium) were evaluated by CT, and it was concluded that this Lipiodol-containing poly(ricinoleic acid-*co*-sebacic acid) polymer is visible on CT, enabling polymer degradation to be potentially monitored noninvasively.^10^ Visualization of this material on both ultrasound and MR has been characterized for this product to determine the optimal polymer composition for conspicuity and biodegradability in vitro and ex vivo for up to 2 weeks.^10^ It was further noted that alteration of the polymer formulation could affect the duration of visualization of these potentially degradable products. Nevertheless, longer-term studies over a time frame of weeks to months needed for clinical markers have yet to be achieved. This includes visualization in the setting of anticipated resorption over time and the safety and biological reaction to these implants. Accordingly, in this study, we evaluate the conspicuity and biodegradability properties of an injectable multimodality polymer over a six-month interval in an in vivo murine model.

## Materials and Methods

Based on clinical need, the compounds for evaluation needed to achieve CT and MR conspicuity lasting between 4 and 24 weeks to cover a wide range of scenarios. Thus, based on a previous study that demonstrated conspicuity and degradation over several weeks,^10^ we chose three potential compounds for evaluation.

### Materials

Materials used for the preparation of Lipiodol implants included PCL (polycaprolactone) with *M*_w_ 14 000 Da; iron (II,III) oxide nanopowder with 50–100 nm particle size (Lot# MKBR5062V) (Sigma Aldrich, Israel); PLGA (poly(lactic-*co*-glycolic acid)) with a 50:50 ratio of lactic acid to glycolic acid and *M*_w_ 17 kDa; PLGA 75:25 with *M*_w_ 18 kDa (PURAC, The Netherlands); lipiodol ultrafluid with an iodine content of 0.49 mg/mL Ch-B:17LU602A (Guerbet, Villepinte, France); and chloroform (CHCl_3_) (BioLab, Israel).

### Polymer Marker Preparation

Each of the three polymers used in this study contained 20% Lipiodol and 0.2% w/w iron oxide based on prior studies documenting the adequate conspicuity of these concentrations on CT and MR, respectively.^10^ The three polymer compounds were composed of Polymer A–PLGA 50:50, Polymer B–PLGA 75:25, and Polymer C–PCL. The polymers were formed into a rod shape of an adequate 1 mm diameter to fit into a biopsy syringe, similar to other commercially available markers used in clinical practice.^11^ Lipiodol, iron oxide, and polymer were added to a 5 mL glass vial, and the mixture was heated to 80 °C for 30 min while hand mixing with a spatula to form a viscous liquid. The molten mixture was then transferred to a hot glass syringe connected to a 17 G needle and cast by pressing through the needle to form black cylindrical rods. The rods were then cut into 2–3 mm implants using an 11-blade scalpel and used for further studies.

### Murine In Vivo Model

A total of nine male BaLB/C OLAHSD mice, 8–9 weeks of age, weighing approximately 20 g, were obtained from Harlan Laboratories (Rehovot, Israel). Each mouse had four markers inserted, the three study polymers and a control substance (the polymer marker without Lipiodol or iron). Mice were housed in cages with free access to food and water. Animal care and the test injections were conducted at a good laboratory practice (GLP)-certified site (Sharett institute SPF unit, Hadassah Medical School), in accordance with the National Institutes of Health guide for the care and use of laboratory animals. The animals were anesthetized with a ketamine–xylazine cocktail: 87.5 mg/kg ketamine (Ketaset, 100 mg/mL, Fort Dodge, Iowa) and 12.5 mg/kg xylazine (20 mg/mL, Biob, France) administrated intramuscularly (IM) at a dose of 5 mL/kg bodyweight. Mice were anesthetized and underwent subcutaneous insertion of polymer markers using a coaxial needle technique (17-gauge) to the back (right and left upper and lower regions, respectively). Three 3 mm × 1 mm markers containing the polymer formulas (A, B, and C) and one control marker, PSA/RA polymer (polymer without contrast agents), were inserted. The location of each polymer marker type was random for each mouse. Euthanasia was achieved by means of carbon dioxide, according to institutional animal care and use committee guidelines.

### Imaging

Imaging was performed at baseline 24 h after implantation and at 2-week scheduled intervals to 6 months (13 scans per mouse). Imaging protocols included serial CT and a final MRI.

### CT Imaging

CT was performed on anesthetized mice, in the prone position, imaged on a 64-detector scanner (Brilliance 64 CT scanner, Philips Medical Systems, Cleveland, OH). Scans were performed with the following parameters: 120 kV, 70 mAs, collimation 64 × 0.625 slice thickness 0.9 mm, increment 0.45 mm, rotation time 0.5 s, and pitch of 0.641. Images were reconstructed using bone and soft tissue algorithms. Follow-up scans were performed every 2 weeks, up to 24 weeks.

### MR Imaging

MR imaging was performed at 6 months for all implanted mice immediately after sacrifice. Mice were placed in the prone position and imaged using a 1.5 T clinical scanner (Avanto, Siemens Healthcare, Belgium) with a 16-channel body coil placed above the animals. Based on prior experience [10], two relevant sequences were acquired: T1 (TR = 2300 ms, TE = 3.05 ms) weighted images, with a 256 mm field of view with a 265 × 265 matrix, a section thickness of 1 mm, and 7.3 mm spacing; and susceptibility weighted images (SWIs), with a 230 × 230 mm matrix on a 230 mm field of view.

### Radiologic/Pathologic Evaluation

**Figure 1:**
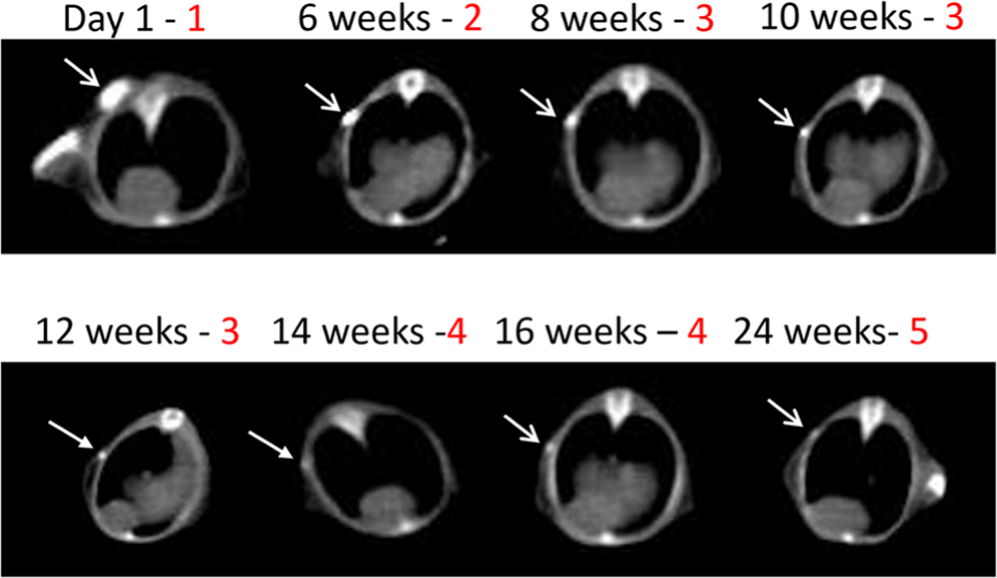
Representative serial axial CT scans of the Lipiodol-containing polymer rod marker with grading of conspicuity over time. Arrows point to polymer C (PCL (polycaprolactone)). Progressive resorption and polymer degradation are noted. Grades of conspicuity (red numbers): 1, baseline; 2, mild decrease; 3, substantial decrease; 4, near-total elimination; and 5, total elimination.

Radiologic–pathologic correlation was performed following mice sacrifice at 6 months. Radiology images of all scans for all mice were reviewed for conspicuity by three readers (SNG, EBD, and HL) in consensus and then subsequently compared over time on mouse-by-mouse and marker-by-marker bases. For the purpose of evaluating clinically acceptable conspicuity of the polymer markers, a five-point scale was used to determine the visualization quality of the markers and the degree of resorption, until deemed not useful clinically. The categories of the scale were as follows: (1) baseline (post insertion); (2) mild resorption (clearly visible); (3) substantial resorption (<50% of initial marker); (4) near-total elimination (barely visible); and (5) total elimination (Figure 1). The baseline, mild, and substantial resorptions were regarded as clinically acceptable, and the fourth and fifth (near-total elimination and total elimination) were not considered adequate for clinical use.

Post sacrifice, gross and histopathology specimens were extracted by resecting the region of the marker placement. The specimens were stained with hematoxylin–eosin. All tissue specimens were examined and evaluated for polymer degradation, iron retention, and inflammatory response.

Representative remaining identified material in the region of marker implantation was sent for biochemical analysis including an iodine/iron analysis by four separate analyses. This included energy-dispersive X-ray analysis (EDX), an X-ray technique used to identify the elemental composition of the materials. The EDX instrument with scanning electron microscopy (Quanta 200, FEI Company) was equipped with an EDAX detector.^12^ To prevent burning, specimens were sputter-coated with palladium at 40 mV for 40 s prior to analysis. Scanning electron microscopy (SEM) (VEGA3, TESCAN) was used for imaging and analyzing the size and morphology of specimens.^13^ The specimens were visualized under a vacuum (upper limit of 6 × 10^–6^ mbar), and images were taken with a beam excitation energy of 20 kV. Next, Fourier transform infrared spectroscopy (FTIR) spectra were recorded with a ThermoScientific FTIR spectrometer (Smart iTR Nicolet iS10 FTIR) with a diamond crystal.^14^ A 5–10 mg sample was placed in a crystal window, and the spectrum was recorded. The scanning range was 400–4000 cm^–1^ with a resolution of 4 cm^–1^. The number of scans for each sample was set to 10. Finally, nuclear magnetic resonance spectroscopy (^1^H NMR) was performed using a Varian Mercury 300 MHz NMR spectrometer. A 10 mg polymer sample was dissolved in 2 mL of deuterated chloroform (CDCl3). The sample solution was transferred to NMR tubes of 5 mm diameter.

### Statistics

For each arm, results were analyzed for each time point comparing the resorption rates for each marker in each mouse, using multivariate ANOVA with t-tests performed for various time points if *p* < 0.05. Additionally, multiple Kaplan–Meier plots with a 95% CI were constructed using the transition between the different grades as the defining endpoint event using MedCalc Statistical Software version 19.5.3 (MedCalc Software Ltd., Ostend, Belgium) for analysis.

Approval for this study was given by the Animal Care and Use Committees of The Hebrew University and the National Council for Animal Experimentation, Israel, in accordance with National Institutes of Health guidelines.

## Results and Discussion

### CT Imaging

**Figure 2:**
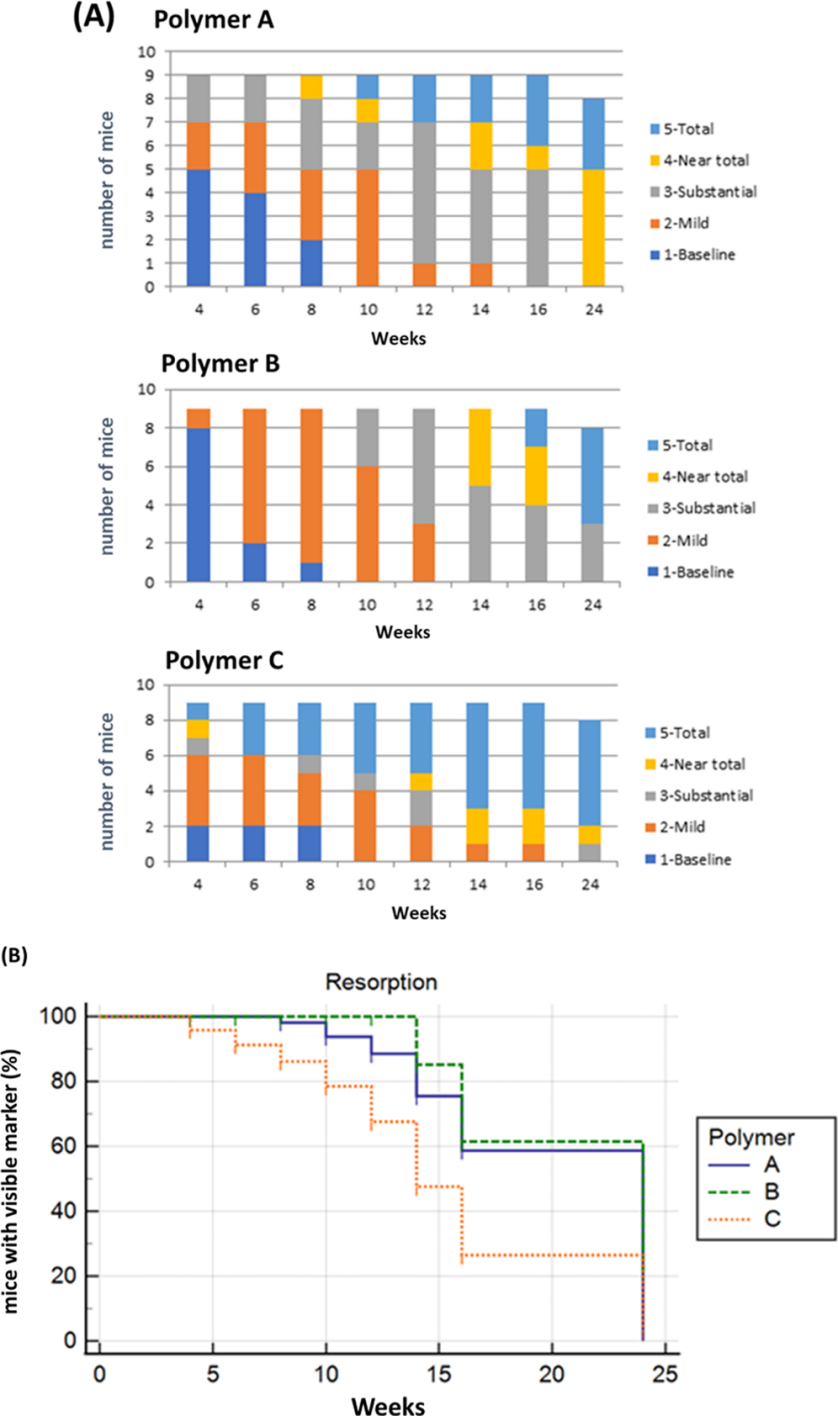
(A, B). Changes in conspicuity over time for three polymers: Polymer A–PLGA 50:50, Polymer B–PLGA 75:25, and Polymer C–PCL. PCL, polycaprolactone; PLGA, poly(lactic-*co*-glycolic acid). (A) Results for all mice graphically. (B) Plot of the % of mice with visible marker over time, for the three polymers. Resorption was defined as reaching a score of 4 (near-total elimination), the point at which visualization is challenging. Statistically significant differences among the polymers are noted (*p* < 0.0001). The categories of the scale were as follows: (1), baseline (post insertion); (2), mild resorption (clearly visible); (3), substantial resorption (<50% of the initial marker); (4), near-total elimination (barely visible); and (5), total elimination.

Eight mice survived to the 6-month study endpoint, with one mouse expiring at week 16 upon anesthetic injection (autopsy showed no pathologic abnormalities or signs of infection or excessive inflammation). Conspicuity on CT, based on the grading system described in the methods section, is summarized in Table 1 and Figure 2a,b. All polymers had similar maximal conspicuity at baseline and showed evidence of degradation over the 6-month study period. However, significant differences in the rate of degradation were observed among the polymers overall (ANOVA; *p* < 0.01), particularly at 8 weeks (*p* < 0.027). Overall, Polymer A began to demonstrate diminution of conspicuity at 4 weeks. However, all markers with this compound remained sufficiently perceivable (grades 1–3) for six weeks. Subsequently, a decline to inadequate levels of visualization (grades 4–5) was seen in 44% by 14 weeks (Figure 2a,b). Polymer B retained excellent conspicuity for at least 8 weeks (grades 1–2), with all animals demonstrating clinical relevance (grades 1–3) for up to 12 weeks. Thereafter, a rapid decline in conspicuity was noted, rendering the markers not clinically usable. Polymer C demonstrated rapid loss of contrast visualization by the 4th week. At six months, there was no visibility of Polymer A in all animals, borderline visibility (grade 3) in 33% of Polymer B, and 11% in Polymer C. As anticipated, the control polymer was not visualized on CT discreetly from the subcutaneous tissues.

**Table 1:** Grading of Degree of Contrast Resorption on CT for the Three Polymers^a^ (Numbers Represent Percentages)

time post implantation										
polymer A (PLGA 50:50)	resorption	day 1	4 weeks	6 weeks	8 weeks	10 weeks	12 weeks	14 weeks	16 weeks	24 weeks
	1. baseline	100	56	44	22	0	0	0	0	0
	2. mild	0	22	33	33	56	11	11	0	0
	3. substantial	0	22	22	33	22	67	44	56	0
	4. near total	0	0	0	11	11	0	22	11	56
	5. total	0	0	0	0	11	22	22	33	33
polymer B (PLGA 75:25)		day 1	4 weeks	6 weeks	8 weeks	10 weeks	12 weeks	14 weeks	16 weeks	24 weeks
	1. baseline	100	89	22	11	0	0	0	0	0
	2. mild	0	11	78	89	67	33	0	0	0
	3. substantial	0	0	0	0	33	67	56	44	33
	4. near total	0	0	0	0	0	0	44	33	0
	5. total	0	0	0	0	0	0	0	22	56
polymer C (PCL)		day 1	4 weeks	6 weeks	8 weeks	10 weeks	12 weeks	14 weeks	16 weeks	24 weeks
	1. baseline	100	22	22	22	0	0	0	0	0
	2. mild	0	44	44	33	44	22	11	11	0
	3. substantial	0	11	0	11	11	22	0	0	11
	4. near total	0	11	0	0	0	11	22	22	11
	5. total	0	11	33	33	44	44	67	67	67

a Numbers represent percentages.

Kaplan–Meier plots for the three polymers were significant, with a 95% CI (*p* < 0.0001, χ^2^ = 23.25) for resorption from grade 3 (substantial resorption, but still clinically visible) to grade 4 (trace visualization, deemed challenging for good visualization in clinical practice) and additionally from grade 4 to grade 5 (trace visualization vs total resorption) (*p* < 0.0001, χ^2^ = 32.58). Transitions from grades 1–2 and 2–3 were not statistically significant (*p* > 0.05).

### MR Imaging

**Figure 3:**
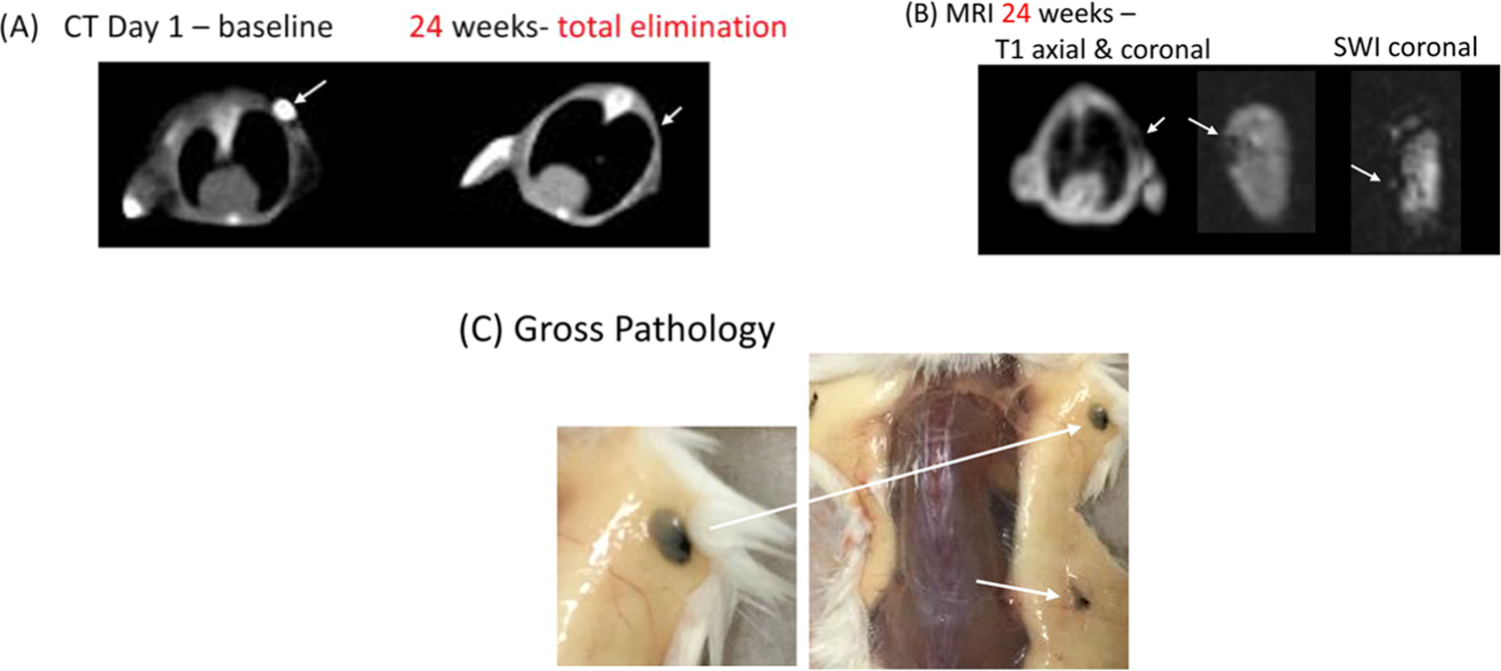
(A–C). CT/MR gross pathologic correlation for Polymer B (PLGA 75:25). (A) CT demonstrates total elimination of the polymer. (B) T1 and SWI show susceptibility artifact, causing a minor local distortion of the image. (C) Gross pathology shows a residual tattooing of the soft tissue with a <1 mm area of the amorphous material (long arrow), with a smaller region of the residual marker noted by the short arrow. PLGA, poly(lactic-*co*-glycolic acid).

At 24 weeks, there was a susceptibility signal at the injection site on T1 and to an even greater degree on the SWI images (Figure 3), without significant distortion of the image at all insertion sites, even in mice where there was total elimination of conspicuity on CT. No MR signal was detected at the injection site of the control marker.

### Radiologic–Pathologic Correlation

**Figure 4:**
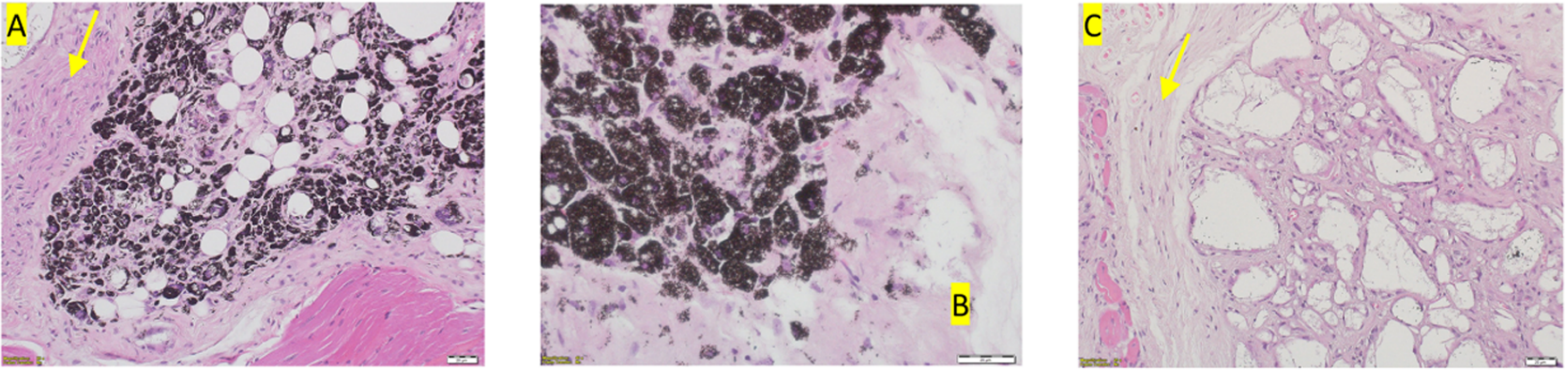
(A–C). Histopathology. Hematoxylin–eosin staining of a sample of Polymer B at 24 weeks. (A) (×20) demonstrates partially resolved polymer residue adjacent to muscle tissue. A small rim of inflammatory cells surrounds the residual polymer (yellow arrow). (B) Higher power (×40) in a different mouse demonstrates a dark granular pigmented appearance caused by iron deposits within macrophages. (C) Control mouse, demonstrating inflammatory response (yellow arrow) surrounding the insertion site, with no apparent residual polymer.

Gross pathologic inspection demonstrated marked dissolving of the markers in all cases where no Lipiodol was detected at CT. Residual “blobs” (2–3 mm) were identified for those four injection sites with a residual CT signal (Figure 3). In the remaining 20 injection sites with total or near-total elimination, there was a 1–2 mm region of a blackened tattoo-like appearance of the soft tissues surrounding the initial implant site that contained clusters of highly pigmented macrophages (Figure 4). Histopathologically, the insertion site was surrounded by several layers of fibroblasts, representing a characteristic inflammatory response similar to that previously reported [10]. Control injection sites had no pockets of macrophages and only limited inflammatory reaction adjacent to fat and muscle.

**Figure 5:**
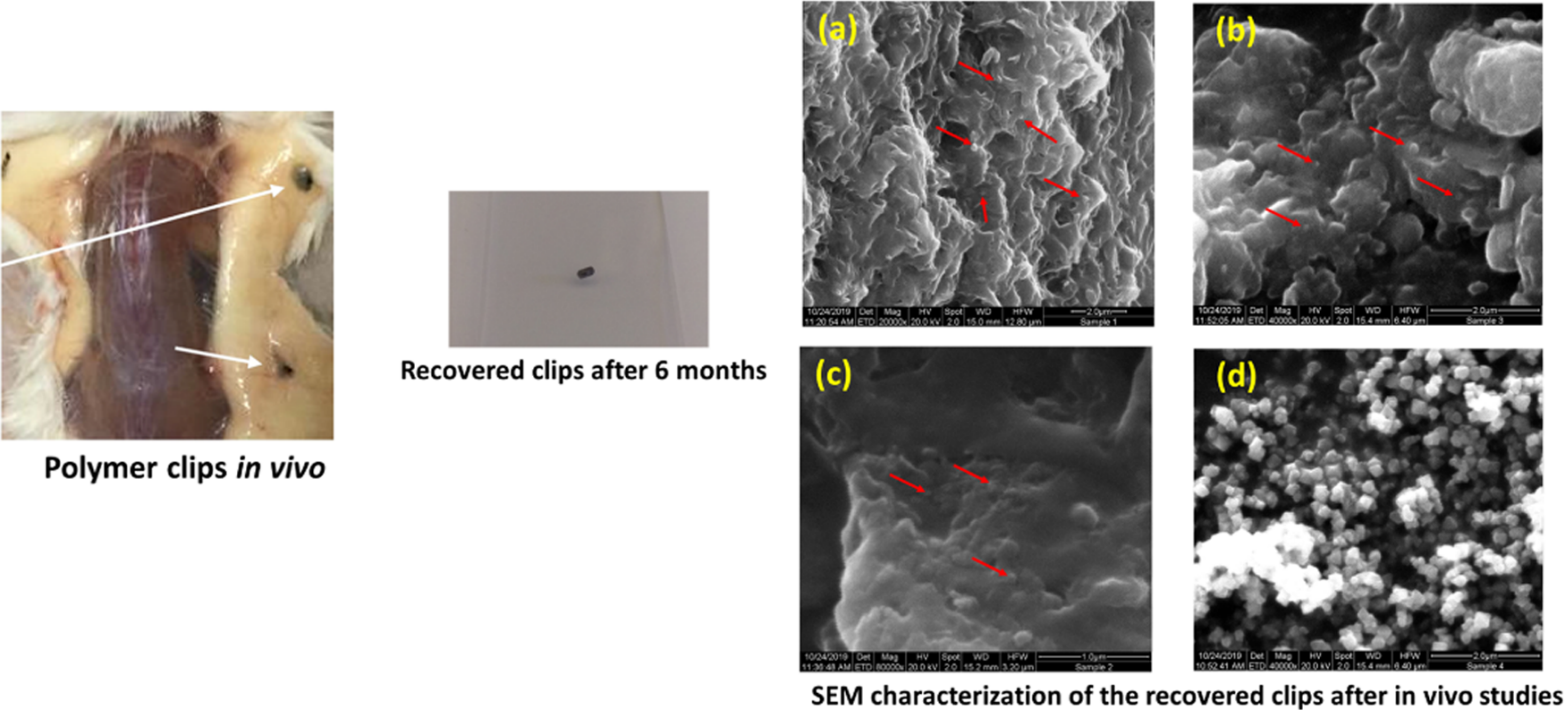
Detection of iron oxide nanoparticles using SEM analysis of the markers after 6 months in vivo study, (a) PCL (Polymer C), (b) PLGA 50:50 (Polymer A), and (c) PLGA 75:25 (Polymer B) markers with Lipiodol and iron oxide after 6 months in vivo degradation. (d) Original iron oxide nanoparticles.

**Figure 6:**
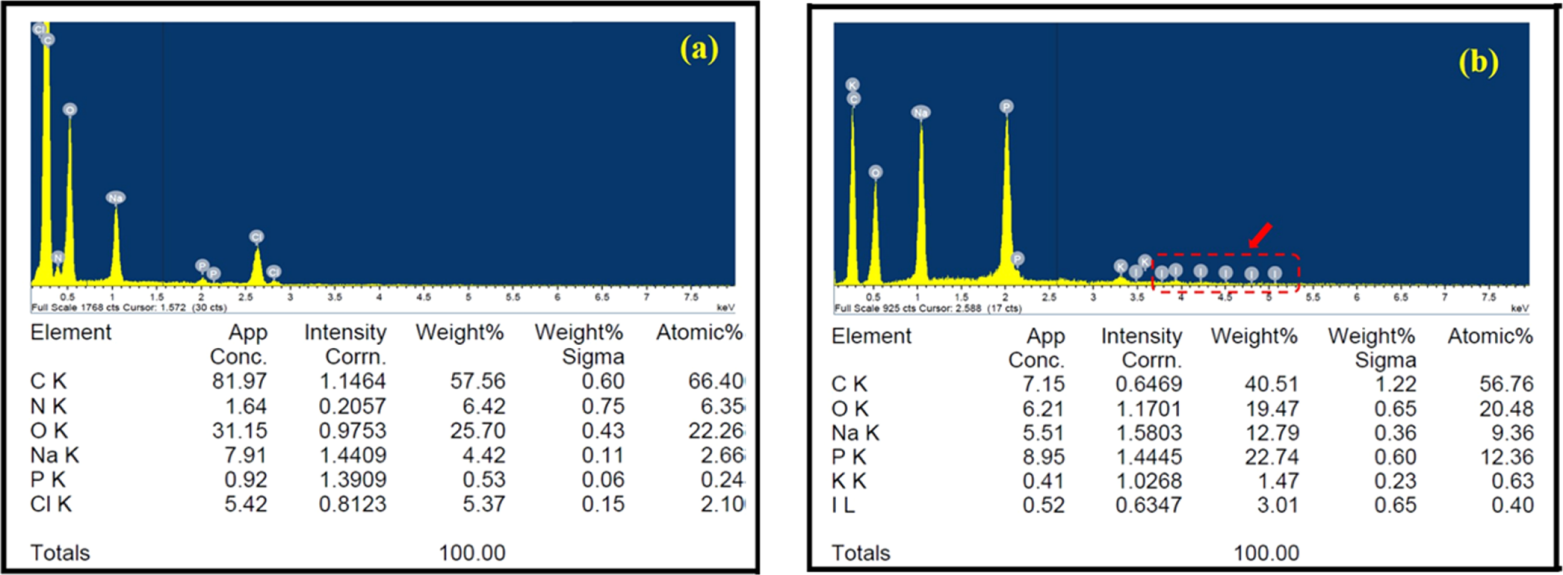
Elemental analysis of PCL (Polymer C) did not detect Lipiodol; however, it was seen in (b) PLGA 50:50 (Polymer A) after 6 months degradation in vivo (red-dotted box and arrow). PLGA 75:25 (Polymer B) polymer markers, not depicted here, demonstrated a profile similar to Polymer A.

The sizes of the markers obtained after 6 months were small and weighed ∼1 mg for Polymer A and Polymer B markers, whereas Polymer C markers weighed 3–5 mg. SEM for iron nanoparticles demonstrated scattered clusters of iron nanoparticles over the surface of the markers (Figure 5). The detection of the iron content by EDX was not possible for any of the markers. Since the amount of marker clips recovered after 6 months in vivo studies was very low, the total iron content in the recovered materials was below the minimal detectable elemental concentration. On the other hand, iodine was detected in Polymer A and Polymer B markers, whereas the Polymer C marker did not show any sign of iodine (Figure 6).

**Figure 7:**
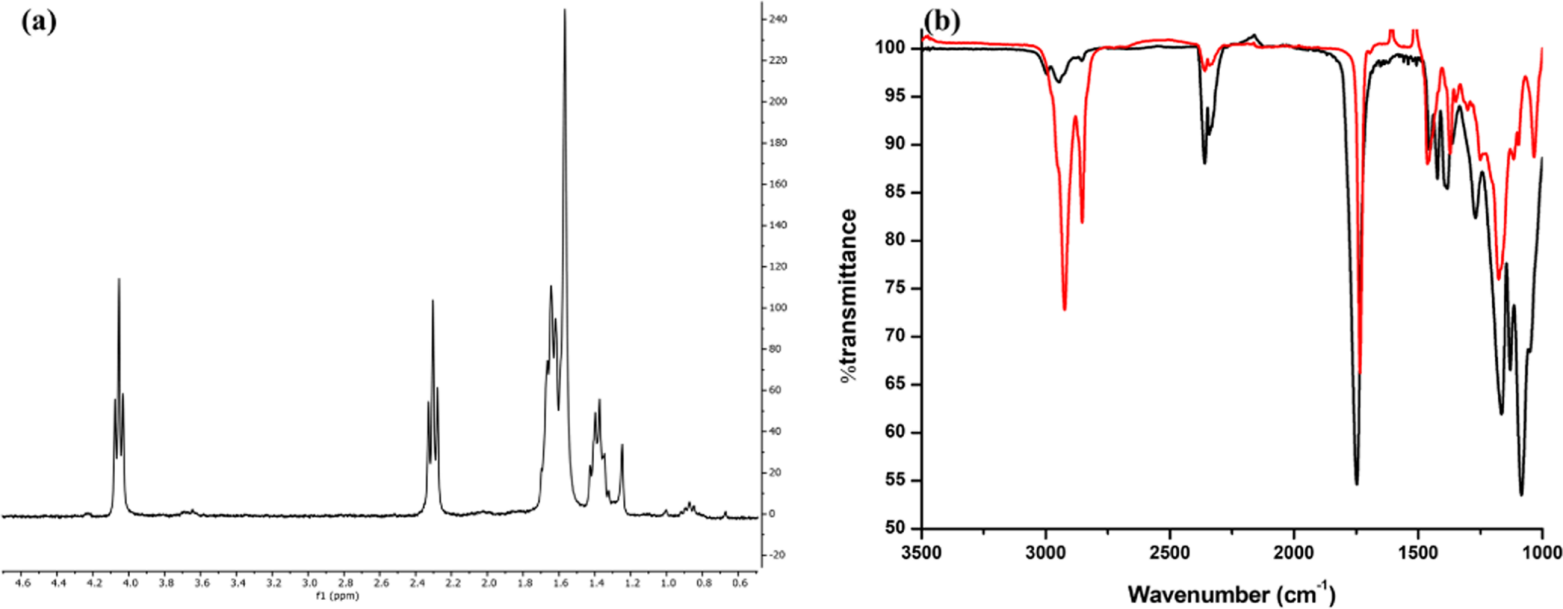
^1^H NMR of (a) PLGA 75:25 (Polymer B) markers with Lipiodol and iron oxide after 6 months in vivo degradation. Polymer B markers showed weak but characteristic peaks of iodine at 0.96 ppm. (b) FTIR analyses of PLGA 50:50 (black) and PLGA 75:25 (red) polymer markers with Lipiodol and iron oxide after 6 months in vivo degradation.

^1^H NMR spectroscopy measurements were performed to identify Lipiodol in the polymer blend. It should be noted that Lipiodol is not a pure compound and hence its exact chemical structure is still unknown. Lipiodol is an iodinated (480 mg iodine/ml) and ethylated ester of poppy seed oil.^15^ However, the signals in its ^1^H NMR spectrum can be used to identify its presence in the polymer clips. The characteristic peaks at 0.96 ppm, 2.25 ppm, and 4.27 ppm have been used to distinguish Lipiodol from the polymers in the clip formulation.^16^ NMR analysis of the markers also confirmed the presence of Lipiodol remaining in the samples after 6 months. However, ^1^H NMR splitting of the polymer and Lipiodol could not be determined in the marker samples because of excess dissolved tissue samples along with iron oxide nanoparticles. Further, for Polymer C markers, no NMR splitting at 0.96 ppm was observed. For Polymer A markers, no characteristic NMR signals were detected, whereas Polymer B markers showed weak but characteristic peaks of Lipiodol at 0.96 ppm (Figure 7).

Finally, FTIR also detected the presence of Lipiodol in the Polymer B markers (Figure 7). Absorption bands at ∼ 1744 cm^–1^ are assigned to C=O stretching, which is similar to the blank PLGA clips.^17^ The FTIR spectra of Lipiodol resemble the FTIR of the poppy seed oil.^18^ Like polymers, the regions of 1700–1800 cm^–1^ for C=O stretching existed in Lipiodol. The visible characteristic absorbance that distinguishes Lipiodol from polymer spectra is only possible at 2854 cm^–1^, which is due to symmetric C–H from phythyl chains (tocopherols) and the band at ∼1371 cm^–1^ for the O–CH_2_ groups^19^ (Figure 7).

In this study, we evaluated three potential degradable polymers formulated and designed to be used as implantable markers visible by multimodality imaging in an in vivo model and note substantially different rates of degradation for different formulations. It is well known that the higher glycolic acid content of the PLGA polymer makes the polymer degrade faster.^20^ However, it was unknown until seen that the PLGA 50:50 degradation also led to a decrease in the sensitivity of the detection of the implants in vivo. On the other hand, Polymer B (PLGA 75:25) achieved the slowest resorption and generated the longest acceptable conspicuity on CT—at least until week 12 post insertion. Hence, it is likely the most suitable for clinical scenarios such as radiation therapy where a 3-month period of visualization on CT and MRI may be considered ideal. Radiation planning for most tumors (such as breast and liver cancers) falls within the 12-week window. This conspicuity may also be beneficial for CT-guided procedures, such as thermal and nonthermal ablation of tumors and image-guided biopsies.^21−23^ While the extended 12-week conspicuity achieved with Polymer B may be beneficial in some clinical instances, some, such as breast surgery, may favor a shorter, 6-week, degradation period, as attained with Polymer A. Yet, the short-lived conspicuity of Polymer C may render it practical only for procedures requiring immediate use (i.e., within days of the insertion).

It is important to note that even after CT conspicuity is lost, MR conspicuity was maintained even at 6 months, likely due to macrophage iron retention.^24^ This is also evident on the gross pathology specimens as a tattooing phenomenon and the scanning electron microscopy results, apparently independent of polymer iodine content. Furthermore, regarding retention of iodine, in Polymers A and B, the iodine-containing Lipiodol bonded, albeit with a decrease in marker size and reabsorption over time. On the contrary, Polymer C was unable to hold the Lipiodol, and thus, the oily Lipiodol leaked out over time from the polymer matrix. This was observed in our study as a decline in the contrast visualization of Polymer C markers on CT by week 4 post implantation. The Lipiodol blends with the three polymers were uniform and stable for months when stored at 4–25 °C. However, when placed in phosphate buffer pH 7.4 at 37 °C, the PCL–Lipiodol blend deformed, after two weeks, into an oil droplet, while PLGA rods retained their shape.

In addition to the polymer composition, the payload may also be hypothetically tailored for specific clinical indications. If a marker is needed to be visible for a long-term medical situation and specifically requires visualization on MRI, then the desired polymer may contain an iron compound. Additionally, iron is beneficial because frank metallic markers will generally have a large artifact on MRI, but theoretically the amount of iron can be altered to allow MRI visualization while minimizing susceptibility artifact. Indeed, iron’s effects are much less pronounced on T1 gradient echo sequences than on SWI images. Thus, if needed, radiation planning may be performed based on MR images using T1 and SWI sequences. However, if the requirement is for a short-term condition and for CT only, a polymer containing only Lipiodol may likely be sufficient.

The histopathologic examination showed dissolution of the polymer marker, with minimal expected inflammatory response, thus adding to the accumulating data on the safety profile of the polymers. Control sites did not demonstrate any significant finding beyond minimal inflammatory changes. Thus, it is likely that the iron deposits seen within macrophages at the insertion site, although they contribute to the visualization on MRI even after 24 weeks, are responsible for the mild inflammation.

Limitations of this study include the fact that we evaluated predominantly CT findings, with only one time point for MRI. Although we have previously documented short-term ultrasonographic conspicuity, long-term US was not performed in this small-animal model as US conspicuity under these superficial conditions is not particularly clinically relevant. Further studies with larger animals may evaluate US conspicuity, as well. Additionally, the use of healthy male mice in this study should not affect the subcutaneous degradation profile of the polymeric implants. Future studies will need to address the rate of degradation in female breast tissues, as well as in pathologic tissues. MR was evaluated only at the final time point, but the fact that the contrast signal remained and early 2-week studies suggest that adequate conspicuity is achievable for this compound (0.2% iron oxide, as in all polymers in this study).

## Conclusions

In conclusion, we were able to identify a group of degradable biocompatible polymers, potentially suitable for clinical use involving CT up to 12 weeks and in MRI for six months. Polymers may be tailored for specific clinical settings, including modality and length of required visualization. Additional studies are planned to refine the formulations of the polymers for optimal balance between visibility and required duration of action versus degree and rate of degradation. Yet, further evaluation is required to examine the long-term conspicuity and degradation properties in larger animal models prior to embarking upon clinical studies.
